# Correction: Physical activity and mental health experiences of people living with long term conditions during COVID-19 pandemic: A qualitative study

**DOI:** 10.1371/journal.pone.0301302

**Published:** 2024-03-22

**Authors:** Leire Ambrosio, Jacqui Morris, Danielle Lambrick, James Faulkner, Eric Compton, Mari Carmen Portillo

## Notice of republication

This article was republished on March 14^th^, 2024, to correct the author list and add Danielle Lambrick and James Faulkner as the third and fourth authors respectively. Please download this article again to view the correct version. The originally published, uncorrected article and the republished, corrected articles are provided here for reference.

## Supporting information

S1 File Originally published, uncorrected article. (PDF)

S2 File Republished, corrected article. (PDF)
