# Supplementary material for: Physical activity and mental health experiences of people living with long term conditions during COVID-19 pandemic: A qualitative study
Source: PLoS One. 2023 Jul 10;18(7):e0285785. doi: 10.1371/journal.pone.0285785 (PMC10332610; doi:10.1371/journal.pone.0285785)
Supplement: S2 Checklist — (DOCX) [file pone.0285785.s002.docx]

STROBE Statement—checklist of items that should be included in reports of observational studies

|  | Item No. | Recommendation | Page  No. | Relevant text from manuscript |
| --- | --- | --- | --- | --- |
| **Title and abstract** | 1 | (*a*) Indicate the study’s design with a commonly used term in the title or the abstract | 1 | Physical activity and mental health experiences of people living with long term conditions during COVID-19 pandemic: a qualitative study |
|  |  | (*b*) Provide in the abstract an informative and balanced summary of what was done and what was found | 1 | Methods. A qualitative study, with in depth videoconference semi-structured interviews were conducted between January and April 2022, with 26 adults living with at least one long term condition in the UK. Data were managed in analytical matrices within Excel and data analysis was conducted using thematic analysis.  Results. Two main themes were developed, explaining how participants managed their physical activity during COVID19 lockdowns, and based on those experiences, what they considered should be in place should another lockdown occur:1) COVID-19 and physical activity: Losses, opportunities and adapting to new formats; and 2) Micro, meso, and macro contexts: creating the right conditions for physical activity support in future pandemics. |
| Introduction | | | |  |
| Background/rationale | 2 | Explain the scientific background and rationale for the investigation being reported | 4 | It is important, therefore, to understand the experiences of people with LTCs regarding PA during COVID-19 to enable future identification of strategies to mitigate the impact of restrictions on health. To our knowledge, there is limited evidence showing the experiences and perspectives of people living with LTCs regarding PA during COVID-19 pandemic in the UK. This study will address this gap. |
| Objectives | 3 | State specific objectives, including any prespecified hypotheses | 5 | Thus, the aim of this study was to explore perceptions and experiences of people with LTCs of the impact of the UK Government physical distancing, self-isolation and/or shielding restrictions on their PA participation during the COVID-19 pandemic, and to identify strategies for mitigating effects in future. |
| Methods | | | |  |
| Study design | 4 | Present key elements of study design early in the paper | 6 | This paper reports a qualitative study, to provide context and meaning to the quantitative data captured initially. Hence, a sequential exploratory approach was applied to qualitatively explore meaning and context. |
| Setting | 5 | Describe the setting, locations, and relevant dates, including periods of recruitment, exposure, follow-up, and data collection | 6-8 | The study population included adults (≥18 years old) living with at least one LTC in the UK (England, Wales, Scotland, and Northern Ireland) who were identified and recruited via social media (Twitter and Facebook) as well as third sector organisations (e.g., Parkinson’s UK, Diabetes UK, or Arthritis Research Action).  In depth videoconference semi-structured interviews were conducted between January and April 2022, via Microsoft Teams or ZOOM. |
| Participants | 6 | (*a*) *Cohort study*—Give the eligibility criteria, and the sources and methods of selection of participants. Describe methods of follow-up  *Case-control study*—Give the eligibility criteria, and the sources and methods of case ascertainment and control selection. Give the rationale for the choice of cases and controls  *Cross-sectional study*—Give the eligibility criteria, and the sources and methods of selection of participants | 6-8 | The study population included adults (≥18 years old) living with at least one LTC in the UK (England, Wales, Scotland, and Northern Ireland) who were identified and recruited via social media (Twitter and Facebook) as well as third sector organisations (e.g., Parkinson’s UK, Diabetes UK, or Arthritis Research Action). Participants able to communicate and understand English, who had a device with internet connection and a microphone and an email address, expressed interest in participation in the project, and could provide informed consent were included in the study. Those that did not meet one or more than one inclusion criteria or reject participating in the study were excluded.  A purposive sampling approach was used [23] with participants from the previous quantitative phase of the mixed method study, who expressed interest in participating in a semi-structured interview. |
|  |  | (*b*) *Cohort study*—For matched studies, give matching criteria and number of exposed and unexposed  *Case-control study*—For matched studies, give matching criteria and the number of controls per case | N/A |  |
| Variables | 7 | Clearly define all outcomes, exposures, predictors, potential confounders, and effect modifiers. Give diagnostic criteria, if applicable | 6-7 | The interview guide was developed to address the research questions ‘how does government-mandated physical distancing/self-isolation/shielding to reduce COVID-19 impact on PA and mental health in adults with LTCs living in the UK?’, what effect does shielding have on the PA and mental health of adults with LTCs living in the UK?’ and ‘what are the barriers and facilitators to PA and/or exercise during self-isolation/shielding for those adults with LTCs living in the UK?’. Considering that COVID-19 related conversation could become a sensitive/uncomfortable conversation for participants, a semi-structured interview guide was conducted considering the language. The interview guide was developed based on a previous literature review in the field [22] and also drew on findings from the previous quantitative phase findings, to explore important issues in more depth. |
| Data sources/ measurement | 8* | For each variable of interest, give sources of data and details of methods of assessment (measurement). Describe comparability of assessment methods if there is more than one group | 8-9 | In depth videoconference semi-structured interviews were conducted between January and April 2022, via Microsoft Teams or ZOOM. Some days before the interview date, the individual received a link via email to join the meeting. Interviews lasted between 45 and 60 minutes although this time varied depending on the participants’ responses. All interviews were recorded, and the audio recordings of the interviews were sent to a professional transcription service of the University of Southampton. |
| Bias | 9 | Describe any efforts to address potential sources of bias | 9 | Next, both researchers collated categories into themes, reviewing and defining them to ensure they provided detailed descriptions of participant experiences and perceptions and their interpretation. |
| Study size | 10 | Explain how the study size was arrived at | 9 | The data analysis began after the first interviews to explore emerging issues as they arose in interviews, and to determine when data saturation had been reached [26]. Data were managed in analytical matrices within Excel spreadsheet. |

Continued on next page

| Quantitative variables | 11 | Explain how quantitative variables were handled in the analyses. If applicable, describe which groupings were chosen and why | N/A |  |
| --- | --- | --- | --- | --- |
| Statistical methods | 12 | (*a*) Describe all statistical methods, including those used to control for confounding | N/A |  |
|  |  | (*b*) Describe any methods used to examine subgroups and interactions | N/A |  |
|  |  | (*c*) Explain how missing data were addressed | N/A |  |
|  |  | (*d*) *Cohort study*—If applicable, explain how loss to follow-up was addressed  *Case-control study*—If applicable, explain how matching of cases and controls was addressed  *Cross-sectional study*—If applicable, describe analytical methods taking account of sampling strategy | N/A |  |
|  |  | (*e*) Describe any sensitivity analyses | N/A |  |
| Results | | | | |
| Participants | 13* | (a) Report numbers of individuals at each stage of study—eg numbers potentially eligible, examined for eligibility, confirmed eligible, included in the study, completing follow-up, and analysed | 10 | A total of 26 individuals living with LTCs were interviewed, 14 male and 12 female participants, aged between 38 and 79 years old. Participant characteristics are presented in Table 2. 20 participants were from England, four were from Scotland and two from Wales. Twenty-four participants had White ethnicity and two had Asian or Asian British – Indian ethnicity. 17 participants were retired, and nine had studies to postgraduate level. Participants were living with different LTCs such as Parkinson’s disease, diabetes mellitus type 2, asthma, heart failure or arthritis (see Table 2 for characteristics). No participant withdrew from the study. |
|  |  | (b) Give reasons for non-participation at each stage | 10 | No participant withdrew from the study. |
|  |  | (c) Consider use of a flow diagram | 11 | Table with included participants and their characteristics |
| Descriptive data | 14* | (a) Give characteristics of study participants (eg demographic, clinical, social) and information on exposures and potential confounders | 11-12 | Overall, data indicate that the precise nature and consequences of the LTCs experienced by the study participants were diverse, but many described their condition and its effects as being changeable. For some this meant gradual worsening of the condition, characterised by increased symptoms, for others, it means difficulties in ability to function, characterised by slowing and fatigue. For a few this meant inability to walk, requiring a wheelchair for mobility, or dependence on spouses for support. Most participants viewed physical activity as essential to managing their condition and their mental health, but their ability to engage in PA fluctuated depending on the status of their condition and on medication management. It was against this context of changeability that the impact of the COVID-19 pandemic affected physical activity. |
|  |  | (b) Indicate number of participants with missing data for each variable of interest | N/A |  |
|  |  | (c) *Cohort study*—Summarise follow-up time (eg, average and total amount) | N/A |  |
| Outcome data | 15* | *Cohort study*—Report numbers of outcome events or summary measures over time | N/A |  |
|  |  | *Case-control study—*Report numbers in each exposure category, or summary measures of exposure | N/A |  |
|  |  | *Cross-sectional study—*Report numbers of outcome events or summary measures | N/A |  |
| Main results | 16 | (*a*) Give unadjusted estimates and, if applicable, confounder-adjusted estimates and their precision (eg, 95% confidence interval). Make clear which confounders were adjusted for and why they were included | N/A |  |
|  |  | (*b*) Report category boundaries when continuous variables were categorized | 12-20 | Data analysis focused on the impact of COVID-19 identified two main themes, explaining how participants managed their physical activity during COVID19 lockdowns, and based on those experiences, what they considered should be in place should another lockdown occur: 1) COVID-19 and PA: Losses, opportunities and adapting to new formats; and 2) Micro, meso, and macro contexts: creating the right conditions for PA support in future pandemics. |
|  |  | (*c*) If relevant, consider translating estimates of relative risk into absolute risk for a meaningful time period | N/A |  |

Continued on next page

| Other analyses | 17 | Report other analyses done—eg analyses of subgroups and interactions, and sensitivity analyses | N/A |  |
| --- | --- | --- | --- | --- |
| Discussion | | | | |
| Key results | 18 | Summarise key results with reference to study objectives | 20-21 | This study explores perceptions and experiences of people living with LTCs of the impact of the COVID-19 lockdown restrictions on their PA participation. These provide information on how people with LTCs managed their condition and generate new understanding of how PA routines changed during the COVID-19 pandemic. Specifically, findings illuminate the effect of lockdown restrictions, and barriers and facilitators to PA during COVID-19 for those living with LTCs. |
| Limitations | 19 | Discuss limitations of the study, taking into account sources of potential bias or imprecision. Discuss both direction and magnitude of any potential bias | 25 | While the study presents interesting results, there are also some limitations that should be recognised. First, the study participants are not geographically diverse as much as would be desired. They are mostly people living in England, so our findings might not be generalizable to the rest of the UK. Secondly, although two researchers were involved in the data analysis to ensure the rigour, their professional backgrounds and personal experiences may have influenced the analysis. Finally, only participants that have a device with internet connection and a microphone, as well as an email address were included. This might limit the transferability of our results. Therefore, it would be necessary to conduct a study that also explores the perception and experiences of people who do not have online resources. |
| Interpretation | 20 | Give a cautious overall interpretation of results considering objectives, limitations, multiplicity of analyses, results from similar studies, and other relevant evidence | 24 | In summary, based on these findings, planning for a future pandemic had to consider solutions at the micro, meso and macro levels. A multisectoral approach which necessitated self-regulation, family and in-person support, and support at community level, with targeted use of volunteers. Information from trustworthy sources including third sector organisations and health professionals was crucial. At the macro level more could be done by the government and public health to foreground the importance of exercise for all, with people with LTCs modelling behaviours for others. |
| Generalisability | 21 | Discuss the generalisability (external validity) of the study results | 25 | The study participants are not geographically diverse as much as would be desired. They are mostly people living in England, so our findings might not be generalizable to the rest of the UK. |
| Other information | |  | | |
| Funding | 22 | Give the source of funding and the role of the funders for the present study and, if applicable, for the original study on which the present article is based | 26 | This study has been funded by the NIHR Applied Research Collaboration Wessex Programme. |

*Give information separately for cases and controls in case-control studies and, if applicable, for exposed and unexposed groups in cohort and cross-sectional studies.

**Note:** An Explanation and Elaboration article discusses each checklist item and gives methodological background and published examples of transparent reporting. The STROBE checklist is best used in conjunction with this article (freely available on the Web sites of PLoS Medicine at http://www.plosmedicine.org/, Annals of Internal Medicine at http://www.annals.org/, and Epidemiology at http://www.epidem.com/). Information on the STROBE Initiative is available at www.strobe-statement.org.
