# Supplementary figures and images for: Physical activity and mental health experiences of people living with long term conditions during COVID-19 pandemic: A qualitative study
Source: PLoS One. 2023 Jul 10;18(7):e0285785. doi: 10.1371/journal.pone.0285785 (PMC10332610; doi:10.1371/journal.pone.0285785)

**
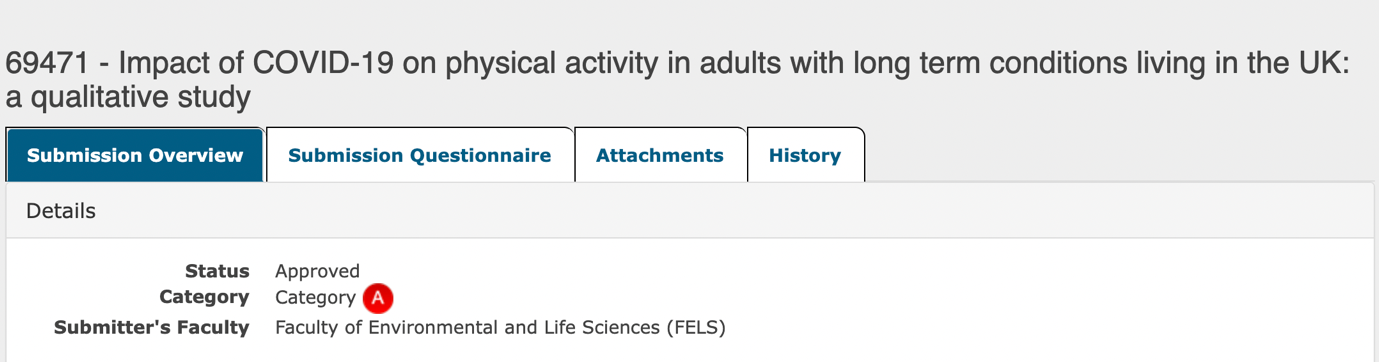
 Supplementary material. Figure 1.** **Ethical Approval**

Supplement: S1 Fig — (DOCX) [file pone.0285785.s003.docx]
