# Supplementary material for: Physical activity and mental health experiences of people living with long term conditions during COVID-19 pandemic: A qualitative study
Source: PLoS One. 2023 Jul 10;18(7):e0285785. doi: 10.1371/journal.pone.0285785 (PMC10332610; doi:10.1371/journal.pone.0285785)
Supplement: S1 Table — (DOCX) [file pone.0285785.s004.docx]

**Supplementary material. Table 1.** Description of the coding tree

| **Codes** |  |
| --- | --- |
| Experiences of living with long term condition   - Initial impact of having a long term condition - Progression of the long term condition - Daily management of the long term condition | |
| Physical activity and long term conditions   - Role of physical activity in managing the long term conditions - Physical activity experienced - Usual physical activity routine - Types of physical activities | |
| Experiences with physical activity   - Previous physical activity experience, before COVID-19 pandemic - Usual physical activity routine in the daily living - Type of physical activity routine | |
| Experience during COVID-19 pandemic   - Impact of COVID-19 pandemic in daily life - Benefits of COVID-19 pandemic - Impact of COVID-19 pandemic in long term condition | |
| COVID-19 pandemic and physical activity   - Impact of COVID-19 pandemic in physical activity - Types of physical activity routines during COVID-19 pandemic - Mode of delivery physical activity during COVID-19 - Impact of delivery mode - Barriers to physical activity during COVID-19 pandemic - Facilitator to physical activity during COVID-19 pandemic - Physical activity service delivery during COVID-19 - Physical activity after COVID-19 pandemic, or after lockdowns ended | |
| Support resources to be physically active during COVID-19   - Third sector services – long term conditions voluntary organization’s role during COVID-19 pandemic - Government, local authorities, role during COVID-19 pandemic - Primary care, NHS, health expert’s role during COVID-19 pandemic - Role of technologies during COVID-19 pandemic - Social and/or community support (groups…) role during COVID-19 pandemic - Social media - social resources available and role during COVID-19 pandemic - Other support resources relevant for the person during COVID-19 pandemic | |
| Mental health experiences   - Impact of physical activity on mental health - Impact of COVID-19 restrictions on mental health - Impact of long term conditions on mental health | |
