# Supplementary material for: Correction: Physical activity and mental health experiences of people living with long term conditions during COVID-19 pandemic: A qualitative study
Source: PLoS One. 2024 Mar 22;19(3):e0301302. doi: 10.1371/journal.pone.0301302 (PMC10959332; doi:10.1371/journal.pone.0301302)
Supplement: S2 File — (PDF) [file pone.0301302.s002.pdf]

PLOS ONE

RESEARCH ARTICLE

Physical activity and mental health experiences of people living with long term conditions during COVID-19 pandemic: A qualitative study

Leire Ambrosio[1]*, Jacqui Morris[2], Danielle Lambrick[3], James Faulkner[4], Eric Compton[5], Mari Carmen Portillo[1]

1 NIHR ARC Wessex, Health and Life Sciences, University of Southampton, Southampton, United Kingdom, **2** School of Health Sciences, University of Dundee, Dundee, United Kingdom, **3** School of Health Sciences, University of Southampton, Southampton, United Kingdom, **4** Department of Sport, Exercise and Health, University of Winchester, United Kingdom, **5** Person with Long Term Conditions, Public and Patient Involvement, Southampton, United Kingdom

* lag1v19@soton.ac.uk

OPEN ACCESS

Citation: Ambrosio L, Morris J, Lambrick D, Faulkner J, Compton E, Portillo MC (2023) Physical activity and mental health experiences of people living with long term conditions during COVID-19 pandemic: A qualitative study. PLoS ONE 18(7): e0285785. https://doi.org/10.1371/journal.pone.0285785

Editor: Fatma Refaat Ahmed, University of Sharjah College of Health Sciences, UNITED ARAB EMIRATES

Received: December 6, 2022

Accepted: May 1, 2023

Published: July 10, 2023

Copyright: © 2023 Ambrosio et al. This is an open access article distributed under the terms of the Creative Commons Attribution License, which permits unrestricted use, distribution, and reproduction in any medium, provided the original author and source are credited.

Data Availability Statement: All relevant data are within the manuscript and its Supporting Information files.

Funding: This study is funded by the National Institute for Health and Care Research ARC Wessex. The views expressed in this publication are those of the authors and not necessarily those of the National Institute for Health and Care

Abstract

Introduction

Regular physical activity is a strategy that is effective in the physical management of long term conditions. The COVID-19 pandemic, led to disruption of physical activity routines for many people with long term conditions. It is important, to understand the experiences of people with long term conditions regarding physical activity during COVID-19 to enable future identification of strategies to mitigate the impact of restrictions on health.

Objective

To explore perceptions and experiences of people with long term conditions of the impact of the UK Government physical distancing restrictions on their physical activity participation during the COVID-19 pandemic.

Methods

A qualitative study, with in depth videoconference semi-structured interviews were conducted between January and April 2022, with 26 adults living with at least one long term condition in the UK. Data were managed in analytical matrices within Excel and data analysis was conducted using thematic analysis.

Results

Two main themes were developed, explaining how participants managed their physical activity during COVID19 lockdowns, and based on those experiences, what they considered should be in place should another lockdown occur:1) COVID-19 and physical activity: Losses, opportunities and adapting to new formats; and 2) Micro, meso, and macro contexts: creating the right conditions for physical activity support in future pandemics.

Research or the Department of Health and Social Care.

Competing interests: The authors have declared that no competing interests exist.

Conclusions

This study provides information on how people with long term conditions managed their condition during the COVID-19 pandemic and generates new understanding of how physical activity routines changed. These findings will be used to inform stakeholder engagement meetings with individuals with long term conditions and local, regional, and national policy makers, to co-produce recommendations that will help people living with long term conditions remain active during and after COVID-19 and other pandemics.

1. Introduction

Long term conditions (LTCs), such as Parkinson's disease, type 2 diabetes mellitus or chronic heart failure, are the leading causes of disability worldwide [1]. In England about 15 million of people live with one or more LTCs [2, 3]. It is estimated that, by 2030, LTCs will account for three quarters of deaths globally, with considerable economic impact due to the costs to the system [4, 5]. More concretely, care for those living with LTCs accounts for a large proportion of NHS costs, amounting to ~70% of the total health care spend in England [3, 6].

LTCs are heterogeneous and affect people in diverse ways. Managing and coping with an LTC is therefore a personal process for individuals [7, 8]. Health and social care professionals should therefore provide person-centred support to enable people to understand and self-manage their condition. To do this they need to deliver tailored strategies that mitigate effects of the condition and promote people to be effective in the long-term management of their condition, whilst accounting for psychological, social and physical factors that influence individuals' lives [9].

Regular physical activity (PA) is a strategy that is effective in the physical management of various LTCs [10] and also improves wellbeing and mental health [11, 12]. PA is defined as any bodily movement produced by skeletal muscles that require energy expenditure [10]. Evidence from systematic reviews and clinical trials shows PA reduces the risk of systemic inflammation, benefits cardiovascular health, increases lung capacity and muscle strength, and improves mental health, among other benefits [13–15]. Many evidence-based clinical guidelines exist to guide PA prescription, for example suggesting that adults should engage in at least 150min/week of moderate to vigorous PA. However, type, intensity and frequency should be adjusted to the needs and abilities of each individual and their circumstances [16].

The global coronavirus disease-2019 (COVID-19) pandemic, led to disruption of PA routines for many people with LTCs [11, 17]. During COVID-19, many governments gradually introduced national and local restrictions that directly influenced peoples' activities, health, and wellbeing. In the UK this included measures such as physical distancing, self-isolation and/or shielding, to limit the spread of COVID-19 for the general population, including those living with LTCs. However, according to a UK national report [18], due to lockdown restrictions people's mobility, mood and independence were negatively affected. Furthermore, physical, and social distancing during COVID-19 has been linked with a 32% decrease in PA, resulting in poorer mental health [17, 19]. There is also evidence showing that adults living with LTCs engaged in less PA during COVID-19 pandemic than before [20]. Therefore, despite UK national guidelines setting out recommendations for adults [21], meeting guideline recommendations was challenging for many people with LTCs or older adults. Also, a recent review [22] showed that government guidance for individuals living with LTCs was to stay at

home and shield during COVID-19 pandemic, without providing any specific guidance on how to become or remain physically active.

It is important, therefore, to understand the experiences of people with LTCs regarding PA during COVID-19 to enable future identification of strategies to mitigate the impact of restrictions on health. To our knowledge, there is limited evidence showing the experiences and perspectives of people living with LTCs regarding PA during COVID-19 pandemic in the UK. This study will address this gap.

Thus, the aim of this study was to explore perceptions and experiences of people with LTCs of the impact of the UK Government physical distancing, self-isolation and/or shielding restrictions on their PA participation during the COVID-19 pandemic, and to identify strategies for mitigating effects in future.

2. Material and methods

2.1 Design

This paper reports a qualitative study, to provide context and meaning to the quantitative data captured initially. Hence, a sequential exploratory approach was applied to qualitatively explore meaning and context.

2.2 Participants

The study population included adults (≥18 years old) living with at least one LTC in the UK (England, Wales, Scotland, and Northern Ireland) who were identified and recruited via social media (Twitter and Facebook) as well as third sector organisations (e.g., Parkinson's UK, Diabetes UK, or Arthritis Research Action). Participants able to communicate and understand English, who had a device with internet connection and a microphone and an email address, expressed interest in participation in the project, and could provide written informed consent were included in the study. Those that did not meet one or more than one inclusion criteria or reject participating in the study were excluded.

A purposive sampling approach was used [23] with participants from the previous quantitative phase of the mixed method study, who expressed interest in participating in a semi-structured interview. To obtain perspectives from a diverse sample, purposeful maximum variation sampling was used with location, demographics, LTCs and reported change in PA and mental health as sampling criteria. Sampling was concurrent with data analysis and continued until the point at which no new ideas emerged, indicating data saturation [24].

2.3 Interview guide development

The interview guide was developed to address the research questions 'how does government-mandated physical distancing/self-isolation/shielding to reduce COVID-19 impact on PA and mental health in adults with LTCs living in the UK?', what effect does shielding have on the PA and mental health of adults with LTCs living in the UK?' and 'what are the barriers and facilitators to PA and/or exercise during self-isolation/shielding for those adults with LTCs living in the UK?'. Considering that COVID-19 related conversation could become a sensitive/uncomfortable conversation for participants, a semi-structured interview guide was conducted considering the language. The interview guide was developed based on a previous literature review in the field [22] and also drew on findings from the previous quantitative phase findings, to explore important issues in more depth. The interview guide was approved by the research team including the patient and public involvement representative (EC, a person living with LTCs) before data collection. See Table 1 for the interview guide.

Table 1. Interview guide.

Experiences living with LTCs	Experiences of physical activity during COVID-19 lockdowns
Tell me about yourself and about how you live and manage your LTC/s and if this affects your life in any way and how	Tell me your views about how has COVID19 impacted upon your physical activity participation?
What role does physical activity play in your life?	How, in your opinion, has COVID-19 lockdowns specifically affect your physical activity?<br>• Related to intensity, duration, and timing of physical activity
What role if any does physical activity play in how you manage your LTC?	Tell me your views about being physically active during COVID-19 this includes the lockdowns but also the time between. What physical activity did you participate in? What resources you had at your disposal?
In your opinion, how does your primary LTC impact upon your physical activity participation?	In hindsight, is there anything you would have done differently, in-terms of your own physical activity during COVID-19 for example, more walking, online classes, reaching out for support from LTC organizations? and also, is there anything that you think you should have done differently?
If you have a second LTC, how, in your opinion, does it impact upon your physical activity participation? Is physical activity more affected due to one condition than the other? How?	In your view, did you have any barrier to engaging in physical activity during COVID-19 (this includes the lockdowns but also the time between)?<br>• If so, what were the barriers to engaging in physical activity?
	Did any changes to your physical activity affect your mental health during COVID-19 (this includes the lockdowns but also the time between)?<br>• If so, tell me your view about how do you think any changes that COVID-19 caused to your physical activity affected your mental health and wellbeing? How have they affected your physical health?
	In your view, if there are further lockdowns or future pandemics, what resources for physical activity do you think should be in place to support people living with LTCs like yours to be physically active and feel safe?
	Tell me your views about who do you think should deliver support for people living with LTCs like you, to be physically active.<br>• In-person, one to one, buddies, online classes/ support, physios, exercise professionals, other people with LTCs
	Any suggestion that you consider would help other people living with one or more than one LTC to be physically active during and also after, COVID-19 lockdowns?

https://doi.org/10.1371/journal.pone.0285785.t001

2.4 Data collection

Participants expressing an interest in this qualitative study were contacted via email by the researcher (LA, a nurse and senior researcher with experience to develop person-centred care to promote a positive living of people with LTCs and experience in qualitative research among other methodologies). They received a participant information sheet, where contact details of the researcher were included so that the individuals could ask any questions about this qualitative study, should they wish. Once they decided to take part, the researcher sent the consent form to the interviewee and a potential interview date was discussed and agreed upon by the participant and the researcher. SafeSend service (software hosted by the University of Southampton) was utilized to transfer research and confidential data across the network securely

encrypted. The written consent form was obtained from all participants in advance of the interview taking place.

In depth videoconference semi-structured interviews were conducted between January and April 2022, via Microsoft Teams or ZOOM. Some days before the interview date, the individual received a link via email to join the meeting. Interviews lasted between 45 and 60 minutes although this time varied depending on the participants' responses. All interviews were recorded, and the audio recordings of the interviews were sent to a professional transcription service of the University of Southampton. Telephone calls were also considered if there were any connection or technological problems or at the interviewee's request.

2.5 Ethical considerations

The study was approved by the University of Southampton Ethics Committee (reference number ERGO: 69471). Participants were informed that their participation was voluntary and that they could withdraw at any time for any reason without affecting their rights. If any discomfort arises regarding the nature of some questions, there was the opportunity to skip questions in the interviews.

For secure storage and in compliance with the Data Protection policy (2018) of the University of Southampton, information regarding the participants as well as hard electronic data (e.g., written consent forms, interviews) was kept on a password-protected computer, strictly confidential. Hence, only one researcher (LA) had access to complete information that could identify individual participants during data collection. After data collection none of the researchers could identify participants.

2.6 Data analysis

Following the six stages of thematic analysis [25] two researchers with prior experience in qualitative researcher conducted the data analysis independently (JM, a physiotherapist and senior rehabilitation researcher with experience in qualitative research with people with LTCs and disabilities and LA) read initial three transcripts and agreed on a coding strategy. LA coded the remaining transcripts. Both researchers reviewed the codes and grouped them into categories relevant to participant experiences of PA during the COVID-19 pandemic. Next, both researchers collated categories into themes, reviewing and defining them to ensure they provided detailed descriptions of participant experiences and perceptions and their interpretation. In addition, findings were shared with the patient and public involvement representative in the project (EC) as the analysis progressed, to incorporate lived experience, evaluating whether the analysis reflected his experiences and making changes according to what he was saying. We also shared findings with the wider PPI group to evaluate whether our interpretation reflected their experiences. Early analysis was deductive and guided by our research questions with emerging ideas coded and incorporated inductively into themes as they were identified in the data. The data analysis began after the first interviews to explore emerging issues as they arose in interviews, and to determine when data saturation had been reached [26]. Data were managed in analytical matrices within Excel spreadsheet.

3. Results

A total of 26 individuals living with LTCs were interviewed, 14 male and 12 female participants, aged between 38 and 79 years old. Participant characteristics are presented in Table 2. 20 participants were from England, four were from Scotland and two from Wales. Twenty-four participants had White ethnicity and two had Asian or Asian British–Indian ethnicity. 17 participants were retired, and nine had studies to postgraduate level. Participants were living

Table 2. Sociodemographic characteristic of participants (n = 26).

Number	Gender	Long term condition
1	Male	Diabetes mellitus type 2 and long term bowel problems
2	Male	Hypertension and COPD
3	Female	Obesity and Parkinson's disease
4	Male	Parkinson's disease
5	Female	Multiple sclerosis
6	Female	Asthma
7	Male	Heart failure
8	Male	Cancer and hypertension
9	Male	Parkinson's disease
10	Female	Heart failure and asthma
11	Female	Parkinson's disease and stroke
12	Male	Parkinson's disease
13	Male	Arthritis
14	Male	Parkinson's disease
15	Female	Rheumatoid arthritis
16	Male	Parkinson's disease and depression
17	Male	Parkinson's disease
18	Female	Depression and COPD
19	Female	Multiple sclerosis
20	Female	Cancer
21	Male	Parkinson's disease
22	Male	Type 2 diabetes mellitus and cancer
23	Female	Cancer
24	Female	Parkinson's disease and asthma
25	Male	Asthma and depression
26	Female	Cancer

https://doi.org/10.1371/journal.pone.0285785.t002

with different LTCs such as Parkinson's disease, diabetes mellitus type 2, asthma, heart failure or arthritis (see Table 2 for characteristics). No participant withdrew from the study. After the 23rd interview, no new ideas were generated from the interviews. Therefore, the authors concluded that the data analysis had reached data saturation. However, three more participants were interviewed to ensure and confirm that here were no new emerging themes.

Overall, data indicate that the precise nature and consequences of the LTCs experienced by the study participants were diverse, but many described their condition and its effects as being changeable. For some this meant gradual worsening of the condition, characterised by increased symptoms, for others, it means difficulties in ability to function, characterised by slowing and fatigue. For a few this meant inability to walk, requiring a wheelchair for mobility, or dependence on spouses for support. Most participants viewed PA as essential to managing their condition and their mental health, but their ability to engage in PA fluctuated depending on the status of their condition and on medication management. It was against this context of changeability that the impact of the COVID-19 pandemic affected physical activity.

Data analysis focused on the impact of COVID-19 identified two main themes, explaining how participants managed their physical activity during COVID19 lockdowns, and based on those experiences, what they considered should be in place should another lockdown occur: 1) COVID-19 and PA: Losses, opportunities and adapting to new formats; and 2) Micro, meso, and macro contexts: creating the right conditions for PA support in future pandemics. See supplementary material for further description of the coding tree.

3.2 Theme 1. COVID-19 and PA: Losses, opportunities and adapting to new formats

Data showed that for many participants, COVID-19 lockdowns meant the sudden loss of opportunities to be active along with the social interaction and motivation that going out to classes, gyms of swimming pools to exercise could provide. However, as the pandemic progressed, many participants found or created opportunities to be active or adapted how they had exercised pre-pandemic to maintain their fitness and mental wellbeing. For some, this was difficult because they lacked motivation or resources. For that group of participants, their more limited activity led to physical changes, worsening their condition, and some weight gain that made returning to regular activity more difficult.

3.2.1 Losses. The losses experienced by participants concerning PA during the pandemic were threefold in nature. Firstly, closures of places within communities to be active, such as classes, gyms and swimming pools, meant that many opportunities to exercise were lost, including being unable to exercise informally with their peers because of social distancing restrictions. The loss of these external motivators meant participants became less active and struggled to find their previous drive for activity.

> *Very difficult. Basically, it's difficult to motivate yourself as well when you're not in the gym. The gym's five minutes away from me, so I used to drive there, park up, go straight in. It's all there isn't it, whereas setting up at home, it takes a bit more energy and planning. I didn't really. . . Couldn't be bothered (participant n°14, Male, living with Parkinson's disease).*

Secondly, the loss of social interaction with others that external exercise opportunities provided was felt strongly and led to feelings of isolation. Many felt vulnerable and feared catching COVID-19, which made them cautious about exercising outside the house even as restrictions were lifted. However, feelings of isolation were counterbalanced by the need to be safe and the sense that these exercise places were no longer safe. This data showed the acceptance of a trade off in which loss of social interaction was the price to be paid for remaining safe.

> I *think the only other thing that I missed was the social aspect because when we used to go to classes, the people who were in the classes, we would maybe go for a coffee afterwards, but because we're all in our own homes, we can't really do that. I think the social aspect was the big thing I missed as well (participant n°17, Male, living with Parkinson's disease).*

> *I'd love to go swimming, but I can't go swimming because the chemo, I don't want to end up getting anything. I think the fear of getting COVID is stronger than the fear of the cancer, if that makes sense. . .I felt secure and looking back, yes, more walking but I was more concerned about getting COVID than anything (participant n°20, Female, living with cancer).*

Participants also commented on having lost the positive impact of exercise on symptoms. Being unable to visit exercise facilities or classes led to worsening symptoms and loss of previously valued benefits of exercise.

> *The swimming runs on to other things. When there was swimming, I was exercising my knee which meant I could walk more, which we used to walk more. It's a vicious circle (participant n° 9, Male, living with Parkinson's disease).*

> *Well, unfortunately, the COVID pandemic really put an end to the kind of exercise that I personally found beneficial. I'm trying to get back into it now, but it's much more difficult. I've*

put on weight—which was not great (participant n° 25, Male, living with asthma and depression).

3.2.2 Finding and creating opportunities. Seeking and creating opportunities to maintain PA was a feature of successful maintenance of activity during lockdown. Despite the challenges and changes imposed by lockdown, many participants could create opportunities for PA within the restrictions. Doing so counterbalanced the loss of freedom to exercise compared to before the pandemic. The determination to remain active despite the imposed lockdown stemmed from the role that many saw PA playing in managing their condition.

Regularly walking was common, an activity allowed by restrictions, and considered safe, because distance to others could be maintained. Walking outdoors was seen as accessible and highly valued because people enjoyed being outdoors in the fresh air in the context of instructions to remain at home. To accommodate the loss of more organised classes and activities, some made an effort to increase the pace and distance of their walking to maintain fitness, and others included dog walking and gardening as types of PA to engage in regularly to maintain fitness. Some incorporated running or cycling as safe activities that were allowed.

It's changed a bit. Instead of going to the gym I would go for a two-and-a-half mile walk, get some cardio workout to keep the circulation going, etc. That takes time and time is something we don't always have. The weather's not always good for walking either! I started doing walking a bit more when I was, during lockdown, on my own (participant n°22, Male, type 2 diabetes mellitus and cancer).

I would do a daily walk, sometimes twice a day, to start and end the day. Then, when I started training with the coach, I would run four days a week, where before I would maybe run two. So that helped (participant n°5, Female, living with multiple sclerosis).

Self-regulation and intrinsic motivation for activity were qualities that supported some participants in creating self-directed opportunities for PA during lockdown. These individuals did this by continuing alone with their exercises. For some with the financial resources, this meant purchasing equipment. The ability to self-regulate their activities and create routines was underpinned by intrinsic motivation to counteract the effects of their condition. Despite expressing desire to exercise with others, these qualities enabled them to overcome the impact of losing organised exercise opportunities. Self-direction in activity was more challenging for those with fewer personal resources, who could not access equipment, or needed social contact or guidance to exercise. These participants recognised that their self-regulation was low and combined with lowered motivation in the absence of social support, led to low activity levels.

For me, being able to go jogging, and doing star jumps and so on enabled me to keep a minimum level of exercise going consistently with the lockdown limitations, which I fully accepted and saw the point of working within, as it were. So, I didn't feel that there was something that should have been done for me to help me to exercise that wasn't actually done, because I didn't need anything (participant n°16, Male, living with Parkinson's disease and depression).

I think if I could have motivated myself, I could have done a lot more activity because we had the time to do it. I think you get into that apathy mode where you just say, 'I can't be bothered.' Every day is the same and, 'I'll do it tomorrow. I'll do it tomorrow.' Then, tomorrow never comes (participant n°23, Female, living with cancer).

3.2.3 Adapting to new formats. Over time organisations moved PA to online formats, and online classes became available to everyone via the media. For some participants, physiotherapy was changed to online delivery. However, participants' ability and willingness to adapt varied. For those who could use technology and had access, engagement with online formats for PA was embraced as a solution, and participants valued the support provided.

The instructor started doing her courses on Zoom; so, eventually, after a couple of months, we did the same courses, but I did it here, and she had 20-odd people on Zoom. We managed it that way; I carried on the exercises in that way (participant n°4, Male, living with Parkinson's disease).

I think it's essential that we maintain and in fact increase online classes and availability to both. It's not just looking at YouTube when you're looking at something that's not personal, you're just following somebody, the online classes, the instructor can actually see and see if you're doing it right and pick up on things. I think other activities which they're trying to do at the moment increase (participant n°21, Male, living with Parkinson's disease)

For others these adaptations were less positive, and the online formats for PA did not match their perceptions of the professional and social support they required.

Actually, standing here jumping up and down in front of a television screen made me feel somewhat stupid. So yes, I'm a group exerciser, but that was my barrier, no people. Yes, I love people (participant n°13, Male, living with arthritis).

Inequalities in access to online formats for exercise was a concern. Participants involved in online groups benefitted from them, but several raised concerns about internet access and computer literacy as significant barriers to engagement for older people and people with disabilities. Therefore, although useful for some, adapting to new formats was not universal, and there was a risk of exclusion from support for activity for some populations. The provision of paper-based resources and in-person support was vital for those unable to access technology.

At the moment, for me—because I'm computer literate and I can use Zoom and I have the technology—it was good. Other people in the group—in the Parkinson's group, that I know, who don't have the technology or the skills, perhaps—because a lot of the people are quite old —I think they suffer more (participant n°4, Male, living with Parkinson's disease).

In summary, the pandemic was disruptive to peoples' PA routines and led to the loss of access to places to exercise and loss of social interaction, which for many had been a central motivating factor for their PA. Loss of opportunities to exercise led to worsening symptoms as the benefits of exercise receded, and motivation waned. However, people with intrinsic motivation, self-regulatory skills, resources and often, access to social support, created their own opportunities to exercise. They did this by finding ways to exercise that did not involve organised classes, walking, running, and creating their own routines. Online exercise classes were accessed by many but were not the solution for all because of accessibility to online and skills required.

3.3 Theme 2. Micro, meso, and macro contexts: Creating the right conditions for PA support in future pandemics

In planning measures to be put in place to ameliorate the impact of future pandemics on the PA of people with LTCs, a spectrum of micro to macro contexts was thought essential. This

meant addressing the impact of the pandemic at the macro (society structures at national or governmental levels), meso (middle groups of organisations like communities, voluntary sector or neighbourhoods) and micro (local individual level e.g. personal networks).

At the micro, individual level, some considered self-regulation of personal activity levels as the primary resource to support activity in the pandemic, especially for walking and running, which required little equipment. Family support, especially for walking, had been crucial for many. Additionally, people needed information on how and when to exercise, but easily finding that was a challenge that should be addressed.

> *I think a lot of support probably could be self-generated if you needed it, but yes, there needs to be some degree of, if you like, marketing of resources around to enable you to access anything that you require. I think part of the responsibility must be on the individual because the information's all there and you just have to use it. I suppose some people aren't able to or aren't capable of finding out about things like that (participant n°3, Female, living with obesity and Parkinson's disease)*

Volunteer services and community groups were highly valued and central to provision of support at the meso level. Local community and third sector organisations were available and accessible resources that could be mobilised again in the future. Efforts made to communicate and stay in touch during lockdown were essential in maintaining mental health and preventing isolation.

Online supports for sharing information and advice and for supporting activity through classes were suggested as future approaches to delivery, through communities and third sector organisations, but with the caveats to accessibility mentioned above considered.

Trustworthiness of advice on PA was important to participants. Volunteers could provide support for PA, but they had to be knowledgeable and able to keep people safe. Several saw health professionals such as general practitioners, physiotherapists, occupational therapists, and exercise professionals with specialist knowledge of their condition as another potential resource at this meso level.

> *Any form of exercise is good for you, but there are exercises with Parkinson's that are specific to them which address the problems of Parkinson's and that tends to happen. The physiotherapists have probably got the experience and the knowledge of what that exercise should be, and they can then cascade it down to your leisure instructors or people instructing on (participant n°21, Male, living with Parkinson's disease)*

Better use of outside spaces and green spaces was another potential approach at the meso level in local communities that could make a difference to individuals by enabling them to exercise locally and safely.

> *I think there should be more provision of outdoor activities for people of different abilities. I think if we look round locally, certainly where I live, yes, you've got a few parks and things but there's not much there. There could have been more things, tai chi classes and things that actually you could do outside, weather permitting or perhaps in some sort of covered—there must have been something (participant n°18, Female, living with depression and COPD)*

At the macro level, local and national government was perceived to be potentially important in providing trustworthy information about PA. However, the guidelines that were provided had been unclear and should be more specific regarding what people who were shielding

could and could not do. PA promotion through advertising and in television programmes were potential options to raise awareness and increase intentions to be active and could be relevant to people who were shielding. Using people with LTCs to provide role models for activity could influence others.

> *The government should put things on television saying 'Exercise', you don't have to go out, you don't have to go to the gym, there's plenty of things you can do in your home. . .You want the message, somebody to deliver the message who people will take notice of and somebody who can be seen to say that the message has made a difference to their life, who is suffering from the same condition that you are (participant n°2, Male, living with hypertension and COPD)*

4. Discussion

This study explores perceptions and experiences of people living with LTCs of the impact of the COVID-19 lockdown restrictions on their PA participation. These provide information on how people with LTCs managed their condition and generate new understanding of how PA routines changed during the COVID-19 pandemic. Specifically, findings illuminate the effect of lockdown restrictions, and barriers and facilitators to PA during COVID-19 for those living with LTCs.

The findings of our study clearly showed that different people deal with COVID-19 pandemic restrictions differently, and their responses differ according to challenges in their everyday life. Overall, losses of opportunities to be active along with the social interaction and outdoor PA activities were identified. Our data showed losses experienced by participants concerning PA were related to the closures of places, and loss of social interaction. Regarding the loss of social interaction, these finding are consistent with previous worldwide studies reporting the increased perceptions of loneliness, psychological stress, and isolation due to COVID-19 pandemic [18, 26, 27]. A challenge of the pandemic was the limitation of direct contact with others [27–29]. As much data form psychological literature shows, contact with others improves mental health [27]. This obviously led to many people having serious problems with maintaining wellbeing. Indeed, loneliness and social isolation is associated with poor quality of life and wellbeing [13, 17, 18]. Also, our data suggest that people with LTCs are a vulnerable group who required particular attention and mental and social support during COVID-19 pandemic. Prioritizing people with multiple LTCs could have avoided the worsening of emotional wellbeing, and further comorbidities, such as obesity, diabetes mellitus, or hypertension among others [17, 22, 30]. Therefore, people with LTCs are an important group to consider when designing and delivering PA guidelines during shielding or social-distancing periods.

According to our findings, the closure of places regarding PA during COVID-19 was reported as a loss. Specifically, closure of classes, gyms and/or swimming pools were identified as a loss of opportunities to exercise, including being unable to exercise informally with others due to social distancing restrictions. Our findings are aligned with previous works [13, 17, 27] showing that restrictions of activities and movement, caused a change in PA behaviours of many people. Literature noted that diverse leisure activities, are important predictors of greater well-being and quality of life [22, 27]. For example, the closure of places meant participants became less active, with more sitting hours and struggled to engage in PA during COVID-19 pandemic. As identified in a recent review [22] accessibility and applicability of PA guidelines, recommendations and/or resources for people living with LTCs during COVID-19 pandemic was variable and unclear. Therefore, based on these findings, more accessible, applicable, and specific PA guidelines and/or recommendations for those living with LTCs is suggested. These

findings have important implications for policy and government recommendations and may assist in refining PA strategies for people with LTCs during COVID-19 pandemic as well as other future pandemics or physical distancing and shielding periods.

In addition, our findings also showed that for some other participants, COVID-19 pandemic created new opportunities with participants being able to adapt to new formats of physical activity support. Despite the challenges, losses and changes imposed by COVID-19 lockdown, many of our participants created their own, new opportunities for PA and adapted to new PA formats, like online classes or walking. It is well known that internet and online resources have played an essential role to remain active and socially connected worldwide during COVID-19 pandemic. However, our findings aligned with previous works, showing that online was not a good and positive option for everyone [11, 31–33]. While online PA formats, such as YouTube were identified as a new and innovative way of being physically active and enjoying exercise when shielding at home, for others online formats were a barrier. Although online resources seem to be the most applicable way to provide PA that aligned with national and international guidelines, during COVID-19 pandemic, internet-based technologies should not be the best option for all population [22, 31, 32]. For instance, for those with digital poverty or technological difficulties, such as no access to electronic devices and/or internet, could make online PA unavailable to them [30]. Therefore, according to this study and other previous works [22, 31] paper-based PA resources in addition to online format seem to be a practical solution to incorporate during COVID-19 pandemic for general population, including people with and without LTCs. These findings have a clear implication in refining government strategies regarding indoor PA guidelines as online resources are not accessible for everyone.

Concerning our findings, planning for a future pandemic seems to require solutions at the micro, meso and macro levels. Our data showed that family and in-person support, and support at community level, with targeted use of volunteers was a key solution for our participants. According to existing evidence, including this study, family support has been crucial during COVID-19 pandemic [27, 34, 35]. It is well known that social support is one of the most common strategies used to deal with the COVID-19 [27]. Particularly, our study showed that family support, including spousal support, has been key to support PA, including walking, during lockdown. Also, volunteer services and community groups were highly valued and central to provision of support during COVID-19 pandemic. However, many of the existing community initiatives and support were paused or were not providing specific PA guidelines and/or recommendations due to COVID-19, and this resulted in a worsening of some physical and emotional wellbeing of those living with LTCs [30, 34]. For future pandemics, providing support to individuals without family online, by telephone or in-person these steps would require planning but were considered by participants as effective. Targeted information or support for PA and social support could be provided through third-sector organisations specific to the LTC. These organisations had often adopted the role of checking on and looking after members during lockdown, were well placed to provide social support and were especially valued by those who were shielding. Therefore, based on this and previous studies [30] information from trustworthy sources including third sector organisations and health professionals is crucial. It is important that personalised and tailored PA guidelines and recommendations strategies reached people with LTCs including appropriate PA prescribing programmes. Also, more effective multidisciplinary referral processes could be conducted to address the complex needs of people with LTCs regarding mobility and PA.

Our findings also showed that more could be done by the UK government and public health to foreground the importance of exercise for all, with people with LTCs modelling behaviours for others. Indeed, limited or at least not enough efforts by the UK health authorities to educate the LTC population about PA guidelines during COVID-19 has been also

showed by other studies [17, 22]. Existing PA guidelines were not always relevant and could have been adapted to be more specific regarding what people who were shielding could do. Therefore, given the numerous physical and mental benefits of increased PA and decreased sedentary behaviour, government and public health authorities should develop and implement interventions that promote safe, efficient, and tailored PA guidelines and/or recommendations if other lockdowns occur. For instance, policy development should consider LTC third sector organisations as well as PA guidelines to design bespoke recommendations for those shielding at home and living with LTCs. Also, a better use of green spaces should be considered to let population including those with LTCs to exercise locally and safely.

In summary, based on these findings, planning for a future pandemic had to consider solutions at the micro, meso and macro levels. A multisectoral approach which necessitated self-regulation, family and in-person support, and support at community level, with targeted use of volunteers. Information from trustworthy sources including third sector organisations and health professionals was crucial. At the macro level more could be done by the government and public health to foreground the importance of exercise for all, with people with LTCs modelling behaviours for others.

Findings emerged in this study are unique showing the effect of lockdown restrictions, as well as the barriers and facilitators to PA during COVID-19 for those living with different LTCs. These findings have important implications for policy and guidelines development. Particularly, findings will be used to inform stakeholder engagement meetings with individuals with LTCs and local, regional, and national policy makers, to co-produce person-centred policy recommendations that will help people living with LTCs remain active during and after COVID-19 and other pandemics.

Strengths of this study are the gender and age variety of the sample size, the analysis by two researchers and public and patient involvement from the beginning of the study. While the study presents interesting results, there are also some limitations that should be recognised. First, the study participants are not geographically diverse as much as would be desired. They are mostly people living in England, so our findings might not be generalizable to the rest of the UK. Secondly, only one participant reported as unable to work and overall, the 36% of the sample have postgraduate educational level. Following the Office for National Statistic [36], our findings might not be representative for those living with LTCs, however we do demonstrate diversity of the sample with respect to other sampling criteria. Thirdly, although two researchers were involved in the data analysis to ensure the rigour, their professional backgrounds and personal experiences may have influenced the analysis. In addition, the interview guide was not pilot tested. However, the authors of this manuscript are fairly certain that the involvement of patient and public involvement mitigated any issues that might influence participants' responses and ability to participate. Finally, only participants that have a device with internet connection and a microphone, as well as an email address were included. This might limit the transferability of our results. Therefore, it would be necessary to conduct a study that also explores the perception and experiences of people who do not have online resources.

5. Conclusions

This study provides information on how people with LTCs manage their condition and generate new understanding of how PA routines changed during the COVID-19 pandemic. Specifically, findings illuminate the effect of lockdown restrictions, as well as the barriers and facilitators to PA during COVID-19 for those living with LTCs. These findings have important implications for policy and guidelines development, particularly for those living with multiple LTCs. The findings of this study will support the development of person-centred policy

recommendations to support and sustain individuals living with LTCs during and after COVID-19 or other pandemics.

Supporting information

S1 Checklist. PLOS ONE clinical studies checklist.
(DOCX)

S2 Checklist. STROBE statement—Checklist of items that should be included in reports of observational studies.
(DOCX)

S1 Fig. Ethical approval.
(DOCX)

S1 Table. Description of the coding tree.
(DOCX)

Acknowledgments

The authors would like to acknowledge the collaboration of all people with long term conditions. Besides, the authors really appreciate the support of third sector organizations during the recruitment process.

Author Contributions

Conceptualization: Danielle Lambrick, James Faulkner, Eric Compton, Mari Carmen Portillo.

Data curation: Leire Ambrosio.

Formal analysis: Leire Ambrosio, Jacqui Morris.

Funding acquisition: Danielle Lambrick, James Faulkner, Mari Carmen Portillo.

Methodology: Leire Ambrosio, Jacqui Morris.

Project administration: Leire Ambrosio.

Supervision: Mari Carmen Portillo.

Validation: Jacqui Morris, Eric Compton.

Writing – original draft: Leire Ambrosio.

Writing – review & editing: Jacqui Morris, Danielle Lambrick, James Faulkner, Eric Compton, Mari Carmen Portillo.

References

1. World Health Organization Noncommunicable diseases; 2021. Available from: https://www.who.int/news-room/fact-sheets/detail/noncommunicable-diseases
2. Stafford M, Steventon A, Thorlby R, Fisher R, Turton C, Deeny S. Briefing: Understanding the health care needs of people with multiple health conditions. 2018. The Health Foundation. Available at: https://www.health.org.uk/sites/default/files/upload/publications/2018/Understanding%20the%20health%20care%20needs%20of%20people%20with%20multiple%20health%20conditions.pdf
3. Naylor C, Parsonage M, McDaid D, Knapp M, Fossey M, Galea A. Long-term conditions and mental health. The cost of co-morbidities. 2012. The King's Fund. Available at: https://www.kingsfund.org.uk/sites/default/files/field/field_publication_file/long-term-conditions-mental-health-cost-comorbidities-naylor-feb12.pdf

4. Mathers CD, Loncar D. Projections of global mortality and burden of disease from 2002 to 2030. PLoS Med. 2006; 3(11):e442. https://doi.org/10.1371/journal.pmed.0030442 PMID: 17132052
5. World Health Organization Health systems respond for non- communicable diseases: time for ambition; 2019. Available from: http://www.who.int/topics/noncommunicable-diseases
6. England NHS. Enhancing the quality of life for people living with Long term conditions. Available at: https://www.england.nhs.uk/wp-content/uploads/2014/09/ltc-infographic.pdf
7. Ambrosio L, Senosiain JM, Riverol M, et al. Living with chronic illness in adults: a concept analysis. J Clin Nurs. 2015; 24(17-18):2357-2367. https://doi.org/10.1111/jocn.12827 PMID: 25951949
8. Rodriguez-Blazquez C, Forjaz MJ, Aya A, Portillo MC, Ambrosio L. Living with Chronic Illness Scale: International validation through the classic test theory and Rasch analysis among Spanish-speaking populations with long-term conditions. Health Expect. 2021; 24: 2065–2077. https://doi.org/10.1111/hex.13351 PMID: 34492734
9. Tobenna DA, Rahkovsky I. Self-Management: A Comprehensive Approach to Management of Chronic Conditions. America J Public Health. 2018; 108: S430–S436.
10. World Health Organisation. 2021 Physical Activity. https://www.who.int/health-topics/physical-activity#tab=tab_1
11. Sallis JF, Adlakha D, Oyeyemi A, Salvo D. An international physical activity and public health research agenda to inform coronavirus disease-19 policies and practices. J Sport Health Sci. 2020.
12. Woods J, Hutchinson NT, Powers SK, Roberts WO, Gomez-Cabrera MC, Radak Z, et al. The COVID-19 pandemic and physical activity. Sport Med Health Sci. 2020; 2(2):55–64. https://doi.org/10.1016/j.smhs.2020.05.006 PMID: 34189484
13. Sallis R, Young DR, Tartof SY, Sallis JF, Sall J, Li Q, et al. Physical inactivity is associated with a higher risk for severe COVID-19 outcomes: a study in 48 440 adult patients. Br J Sports Med. 2021; 55(19): 1099–1105 https://doi.org/10.1136/bjsports-2021-104080 PMID: 33849909
14. Buitrago-Garcia D, Egli-Gany D, Counotte MJ, et al. Occurrence and transmission potential of asymptomatic and presymptomatic SARS-CoV-2 infections: a living systematic review and meta-analysis. PLoS Med. 2020; 17:e1003346. https://doi.org/10.1371/journal.pmed.1003346 PMID: 32960881
15. Piercy KL, Troiano RP, Ballard RM, et al. The physical activity guidelines for Americans. JAMA 2018; 320:2020–2028. https://doi.org/10.1001/jama.2018.14854 PMID: 30418471
16. Siedler MR, Lamadrid P, Humphries MN, Mustafa RA, Falck-Ytter Y, Dahm P, et al. The quality of physical activity guidelines, but not the specificity of their recommendations, has improved over time: a systematic review and critical appraisal. Appl Physiol Nutr Metab. 2021; 00: 1–12 (0000).
17. Faulkner J, O'Brien W, McGrane, Wadsworth D, Batten J, Askew CD, Badenhorst C, et al. Physical activity, mental health and well-being of adults during initial COVID-19 containment strategies: A multi-country cross-sectional analysis. J Sci Med Sport. 2021; 320–326. https://doi.org/10.1016/j.jsams.2020.11.016 PMID: 33341382
18. Age UK. Impact of COVID-19 on older people mental health and physical health: one year on. 2021. Available at: https://www.ageuk.org.uk/globalassets/age-uk/documents/reports-and-publications/reports-and-briefings/
19. Meyer J, McDowell C, Lansing J, et al. 2020. Changes in physical activity and sedentary behaviour due to the COVID-19 outbreak and associations with mental health in 3,052 US adults. Cambridge Open Engage. 2020.
20. Rogers NT, Waterlow NR, Bringle H, Enria L, Eggo RM, Lees S, et al. Behavioral Change Towards Reduced Intensity Physical Activity Is Disproportionately Prevalent Among Adults With Serious Health Issues or Self-Perception of High Risk During the UK COVID-19 Lockdown. Front Public Health. 2020; 8: 1–12.
21. UK Chief Medical Officers' PA guidelines. 2019. Available from: https://assets.publishing.service.gov.uk/government/uploads/system/uploads/attachment_data/file/832868/uk-chief-medical-officers-physical-activity-guidelines.pdf
22. Ambrosio L, Lambrick D, Faulkner J, Portillo MC. Accessibility and applicability of physical activity guidelines and recommendations for adults living with long term conditions during COVID-19. Int J Environ Res Public Health. 2022; 24: 1–17. https://doi.org/10.1080/09603123.2022.2066071 PMID: 35466819
23. Miles MB, Huberman AM, Saldaña J. Qualitative data analysis. A methods sourcebook, 3rd ed. Sage Publications. 2014.
24. Malterud K, Siersma VD, Guassora AD. Sample Size in Qualitative Interview Studies: Guided by Information Power. Qual Health Res. 2016; 26(13): 1753–1760. https://doi.org/10.1177/1049732315617444 PMID: 26613970
25. Braun V, Clarke V. Using thematic analysis in psychology. Qual Res Psyc. 2006; 3(2): 77–11.

26. Pietrabissa G, Simpson SG. Psychological Consequences of Social Isolation During COVID-19 Outbreak. Front Psychol. 2020: 11; 1–4. https://doi.org/10.3389/fpsyg.2020.02201 PMID: 33013572

27. Maison D, Jaworska D, Adamczyk D, Affeltowicz D. The challenges arising form the COVID-19 pandemic and the way people deal with them. A qualitative longitudinal sutyd. PLoS One. 2021; 11: 1–17

28. Kumar N, Gupta R, Kumar H, Mehta S, Rajan R, Kumar D, et al. Impacto f home confinement during COVID-19 pandemic on Parkinson's disease. Parkinsonism Relat Disord. 2020; 80: 32–34. https://doi.org/10.1016/j.parkreldis.2020.09.003 PMID: 32937224

29. Anghelescu BA, Bruno V, Martino D, Roach P. Effects of the COVID-19 pandemic on Parkinson's disease: a single-centered qualitative study. Canadian J Neurol Sci. 2022; 49(2): 171–183. https://doi.org/10.1017/cjn.2021.70 PMID: 33843516

30. Soilemezi D, Roberts H, Navarta-Sanchez MV, Kunkel D, Ewings S, Reidy C et al. Managing Parkinson's during the COVID-19 pandemic: Perspectives from people living with Parkinson's and health professionals. J Clin Nurs. 2022; 00(1): 1–12.

31. Pan Q, Reichert F, de la Torre J, Law N. Measuring digital literacy during the COVID-19 pandemic: experiences with remote assessment in Hong Kon. Educ Meas Issues Pract. 2022; 0(0): 1–5.

32. Schirinzi T, Ceronni R, Di Lazzaro G, Liguori C, Scalise S, Bovenzi R, et al. Self-reported needs of patients with Parkinson's disease during COVID-19 emergency in Italy. Neurol Sci. 2020; 41: 1373–1375 https://doi.org/10.1007/s10072-020-04442-1 PMID: 32363506

33. Norman C, Wildman JM & Sowden S. COVID-19 at the deep end: qualitative interview study of primary are staff working in the most deprived areas of England during COVID-19 pandemic. Int J Environ Res Public Health. 2021; 18(6): 8689.

34. Hart JL, Turnbull AE, Oppenheim IM, Courtright KR. 2020. Family-Centered Care During the COVID-19 Era. J Pain Symptom Manage. 2020: 60(2); e93 –e97. https://doi.org/10.1016/j.jpainsymman.2020.04.017 PMID: 32333961

35. Merritt RK, Hotham S, Graham L, Schrag A (2018). The subjective experience of Parkinson's disease: a qualitative study in 60 people with mild to moderate Parkinson's in 11 European countries. Eur J Pers Cent Healthc. 2018; 6(3).

36. Office for National Statistics. People, population and community. Available from: https://www.ons.gov.uk/peoplepopulationandcommunity
